# Infrared neural stimulation markedly enhances nerve functionality assessment during nerve monitoring

**DOI:** 10.1038/s41598-023-31384-3

**Published:** 2023-03-16

**Authors:** Graham A. Throckmorton, Wesley Thayer, E. Duco Jansen, Anita Mahadevan-Jansen

**Affiliations:** 1 Department of Biomedical Engineering, Vanderbilt University, 5824 Stevenson Center, Station B, Box 351631, Nashville, TN 37235-1631 USA; 2 Vanderbilt Biophotonics Center, 410 24th Ave. South, Nashville, TN 37232 USA; 3 Department of Plastic Surgery, Vanderbilt University Medical Center, 1211 Medical Center Drive, Nashville, TN 37232 USA; 4 Department of Neurological Surgery, Vanderbilt University Medical Center, 1161 21St Avenue, Nashville, TN 37232-2380 USA; 5 Department of Otolaryngology-Head and Neck Surgery, Vanderbilt University Medical Center, 1211 Medical Center Drive, Nashville, TN 37232 USA; 6 Department of Surgery, Vanderbilt University Medical Center, 1211 Medical Center Drive, Nashville, TN 37232 USA

**Keywords:** Biophotonics, Evoked potentials

## Abstract

In surgical procedures where the risk of accidental nerve damage is prevalent, surgeons commonly use electrical stimulation (ES) during intraoperative nerve monitoring (IONM) to assess a nerve’s functional integrity. ES, however, is subject to off-target stimulation and stimulation artifacts disguising the true functionality of the specific target and complicating interpretation. Lacking a stimulation artifact and having a higher degree of spatial specificity, infrared neural stimulation (INS) has the potential to improve upon clinical ES for IONM. Here, we present a direct comparison between clinical ES and INS for IONM performance in an in vivo rat model. The sensitivity of INS surpasses that of ES in detecting partial forms of damage while maintaining a comparable specificity and sensitivity to more complete forms. Without loss in performance, INS is readily compatible with existing clinical nerve monitoring systems. These findings underscore the clinical potential of INS to improve IONM and surgical outcomes.

## Introduction

Iatrogenic nerve injuries (INI) have plagued surgical outcomes across all specialties^1–6^. Between 450,000 and 600,000 INIs occur each year in the United States alone^2,7–9^. In some cases like prostatectomies and mastectomies, the prevalence of INIs is reported to be as high as 85% and 60% respectively^10,11^. The deleterious complications due to INI can range from numbness and loss of sensation to chronic pain and paralysis^6,12–16^. Moreover, INIs are also a common source of medicolegal litigation with 60% of INI complications during thyroid surgery leading to malpractice lawsuits and 82% of cases of spinal accessory nerve injury resulting in patient compensation^17,18^.

Diagnosis of INI is largely dependent on the surgeons’ awareness of the injury and its symptoms that develop postoperatively^19^. Consequently, intraoperative nerve monitoring (IONM) has been used since the late 1970s to alert surgeons to the onset of nerve damage and lower the incidence of INIs^15,20–23^. IONM seeks to preserve peripheral nerve function through electrical stimulation (ES) of at risk nerves throughout surgery and examining any changes in the amplitude and latency of the evoked signals that are indicative of damage. By assessing nerve functionality throughout a surgical procedure, the risk of INI is greatly reduced and timely interventions can be made if damage occurs. Because IONM relies on ES, however, IONM suffers from several ES-based shortcomings.

First, ES requires contact with tissue to excite action potentials. Thus, changes in the degree of contact between the electrode and tissue can lead to misrepresentative evoked responses^24^. Additionally, high frequency artifacts have long hindered proximal ES and electrophysiological recordings due to the superposition of the artifact onto the evoked signal. In the context of IONM, ES artifacts can often obscure the onset and alter magnitude of the evoked response making it impossible to accurately calculate the amplitude and latency needed to detect and prevent further damage^25^. Lastly, ES is prone to current spread in which unconfined charge is distributed throughout the adjacent tissue^26–30^. As a result, ES excites distant neural tissue beyond the intended target leading to potential misdiagnosis of nerve functionality and viability. Currently, surgeons are still searching for better stimulation techniques to improve the spatial resolution of IONM^31,32^.

Infrared neural stimulation (INS) is a label-free, optical method to excite neural tissue using pulsed infrared light ($\lambda = 1440 - 1550\;{\text{nm}}$ and $\lambda = 1850 - 2120 \;{\text{nm}})$^33–37^. During INS, absorbed infrared light initiates action potentials via a thermally-mediated transient change in cell membrane capacitance which generates a depolarizing current^38–40^. Due to its mechanism, INS is confined to small volumes dictated by the laser spot size and wavelength-dependent optical penetration, providing a high degree of innate spatial specificity^29,41,42^. Consequently, the spatial precision of INS has been shown to selectively stimulate specific nerve fascicles, ocular dominance columns, and substructures of embryonic quail hearts among other targets^29,34,43^. Additionally, owing to its unique mechanism, INS does not produce stimulation artifacts enabling simultaneous stimulation and recording in neighboring areas^43,44^. Unlike its electrical counterpart, INS does not require contact with the target tissue. Human feasibility studies have shown that INS is a safe and effective means of clinical neurostimulation in the acute, intraoperative setting^41^.

Here, we demonstrate the application of INS as a potential clinical tool for IONM in an in vivo sciatic nerve model. To directly compare INS to standard clinical ES, nerves were monitored using both modalities before and after partial or complete transection, crush, or stretch. In examining varying degrees of the three most prevalent INIs, INS outperforms ES exhibiting a higher sensitivity to less severe forms of damage due to its spatial selectivity. The efficacy of INS during IONM is also consistent across benchtop and clinical nerve monitoring systems. Improved sensitivity to less severe forms of injury could alert surgeons to the onset of damage earlier preventing further trauma and enabling timely interventions.

## Results

To directly compare ES and INS, an in vivo rat sciatic nerve preparation was utilized to examine each techniques’ ability to detect different forms and degrees of nerve injury (Table 1). The rat sciatic nerve along with its trifurcations share similar diameters (0.25–0.90 mm) to that of human nerves such as the recurrent laryngeal (0.71–2.0 mm) and facial nerve (1.1–2.6 mm) which are commonly monitored intraoperatively^45–47^. Baseline compound muscle action potential (CMAP) amplitude and latency values were acquired for both ES and INS at the beginning of each trial from either the common peroneal or tibial nerve branch. After ~ 10 min, another set of amplitude and latency measurements were obtained with both techniques to ensure reproducibility of baseline, healthy values. To assess each modality’s sensitivity to injuries of varying severity, the interrogated nerve was then partially damaged with a transection, crush, or stretch injury and then stimulated again. These three types of injury are the most common types of INIs and are representative of all three Seddon classifications^48,49^. Lastly, the nerve was completely damaged and stimulated again to acquire amplitude and latency values. As the current clinical standard, a ≥ 50% loss in baseline amplitude and a ≥ 10% increase in the baseline latency serve as the thresholds for neural damage detection^50,51^. Once completed, the entire protocol was repeated for the remaining sciatic nerve branch.

**Table 1:** Examined nerve injuries, methods, extent, and Seddon classification*.

Injury type	Method	Partial injury		Complete injury	
		Extent	Seddon classification	Extent	Seddon classification
Transection	Razor blade via nerve cutting guide	Half the diameter of the nerve severed	Neurotmesis	Cut through entirety of the nerve	Neurotmesis
Crush	Calibrated hemostats	Half of the nerve diameter crushed	Axonotmesis	Crushed entire diameter of the nerve	Axonotmesis
Stretch	Hook electrodes mounted to micromanipulator	Nerve stretched to an average strain of:$\varepsilon =8.6\pm 1.6\%$	Neuropraxia	Stretched nerve to an average strain of:$\varepsilon =13.4\pm 3.6\%$	Neuropraxia

*Seddon^58^ and Seddon et al.^59^.

### Partial nerve transections are more reliably detected by infrared neural stimulation

To determine whether INS offers any benefit in identifying transections, nerves were partially and completely severed while using INS and ES for IONM. A 3D printed nerve cutting guide was used to cut through approximately half the nerve’s diameter (41–59%) perpendicular to its long axis with a razor blade. The nerve was entirely severed for the complete form of transection injury. After baseline values were collected, the undamaged nerves were restimulated to ensure values were consistent (designated as the ‘Healthy’ condition in all figures) and to obtain a specificity.

**Figure 1:**
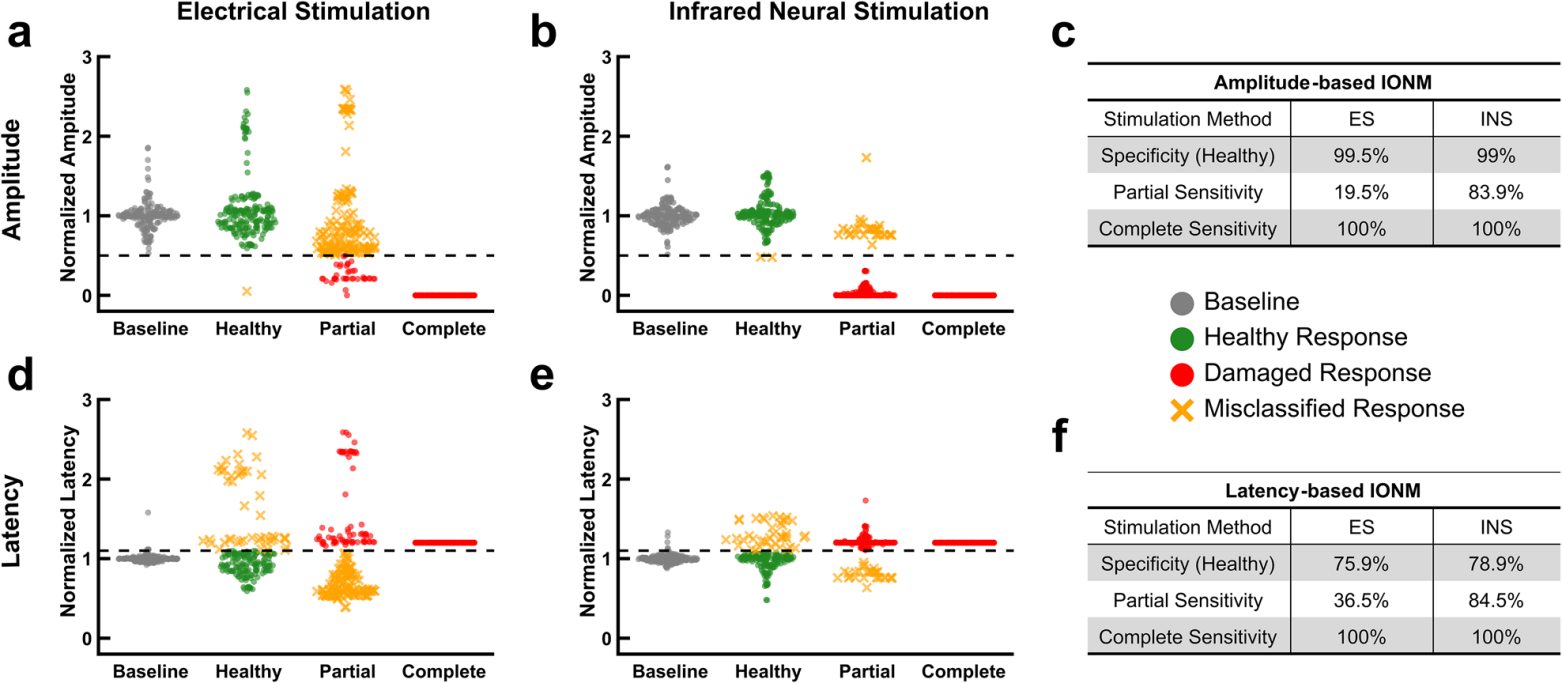
Infrared neural stimulation is more sensitive to partial nerve transections. (**a)** Normalized CMAP amplitudes resulting from ES in baseline, healthy, partial transection, and complete transection conditions. Black dashed line represents the amplitude damage threshold (50% decrease). (**b**) Normalized CMAP amplitudes resulting from INS in baseline, healthy, partial transection, and complete transection conditions. (**c**) The sensitivity and specificity for the amplitude-based IONM approach to transection injuries. (**d**) Normalized CMAP latencies resulting from ES in baseline, healthy, partial transection, and complete transection conditions. Black dashed line represents the latency damage threshold (10% increase). If no CMAP was evoked latency was set to 1.2 for ease of visual interpretation. (**e**) Normalized CMAP latencies resulting from INS in baseline, healthy, partial transection, and complete transection conditions. (**f**) Sensitivity and specificity for the latency-based IONM approach to transection injuries. Specificity in all cases was calculated using the ‘Healthy’ category of responses. All data is normalized to the mean baseline values for each individual nerve. $n = 10$ nerves for all data sets (5 rats).

The specificity of INS and ES are nearly equivalent for both amplitude and latency-based approaches (Fig. 1c, f). However, ES exhibits a broader amplitude distribution in the healthy condition (Fig. 1a) than INS whose healthy distribution nearly replicates that of the baseline values (Fig. 1b). Moreover, ES largely fails to indicate the presence of a partial transection (Sensitivity = 19.5%) while INS detects the injury the majority of the time (Sensitivity = 83.9%; Fig. 1a, b). This is also consistent for latency-based IONM (Fig. 1d, e). Trials in which INS misclassified partial transection as healthy are a result of its spatial selectivity.

All nerves were damaged distally to the point of trifurcation and stimulated proximally. Trials in which INS did not detect nerve partial transections activated fascicles whose distal segments remained in continuity rather than activating fascicles whose distal segments had been transected (Fig. S1a). This was confirmed by translating the fiber across the nerve and observing the corresponding loss of CMAPs even at higher radiant exposures (Fig. S1a). Both methods exhibit equal sensitivities to complete transections as no action potential propagation is possible.

### Infrared neural stimulation is more sensitive to crush injuries

**Figure 2:**
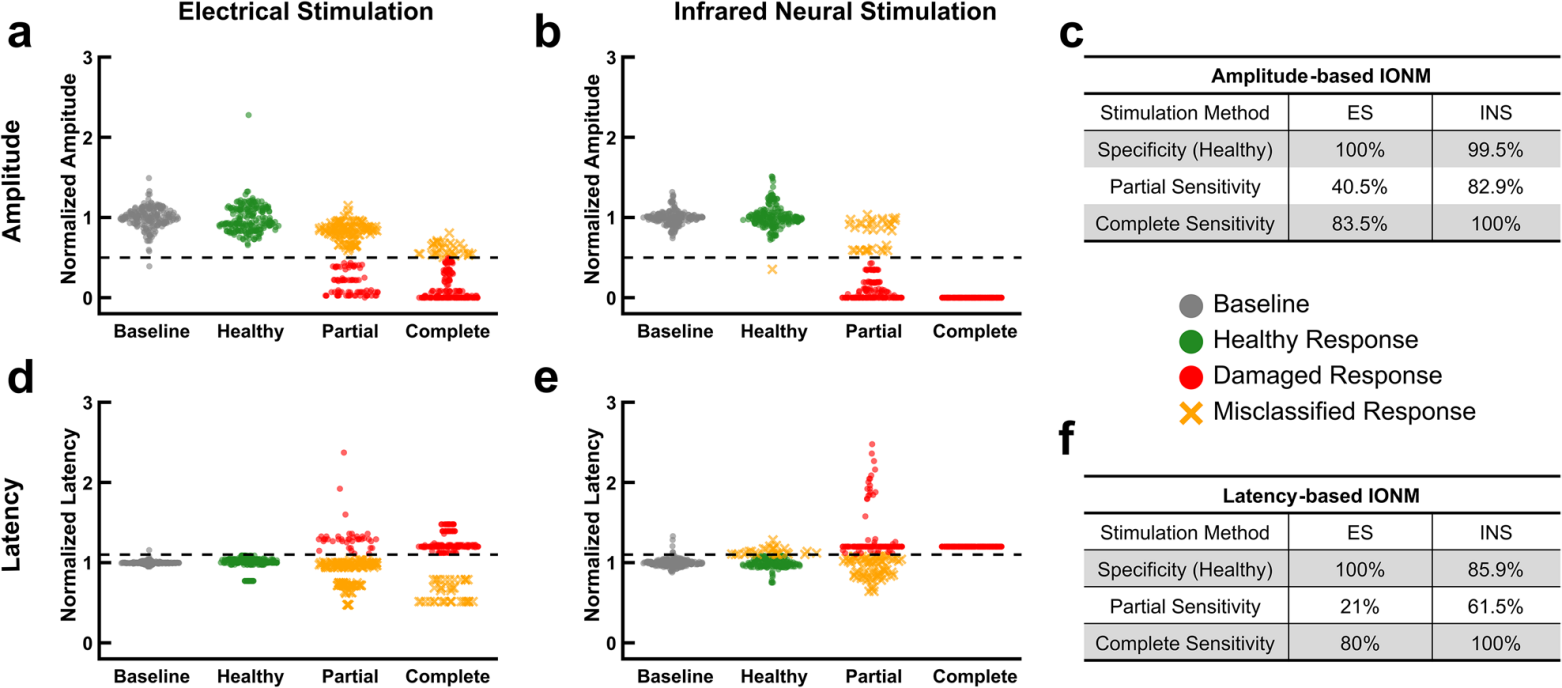
Infrared neural stimulation more readily detects crush injuries. (**a**) Normalized CMAP amplitudes resulting from ES in baseline, healthy, partially crush, and complete crush conditions. Black dashed line represents the amplitude damage threshold (50% decrease). (**b**) Normalized CMAP amplitudes resulting from INS in baseline, healthy, partially crush, and complete crush conditions. (**c**) Sensitivity and specificity for the amplitude-based IONM approach to crush injuries. (**d**) Normalized CMAP latencies resulting from ES in baseline, healthy, partial crush, and complete crush conditions. Black dashed line represents the latency damage threshold (10% increase). If no CMAP was evoked latency was set to 1.2 for ease of visual interpretation. (**e**) Normalized CMAP latencies resulting from INS in baseline, healthy, partial crush, and complete crush conditions. (**f**) Sensitivity and specificity for the latency-based IONM approach to crush injuries. Specificity in all cases was calculated using the ‘Healthy’ category of responses. All data is normalized to the mean baseline values for each individual nerve. $n = 10$ nerves for all data sets (5 rats).

Crush injuries were inflicted by transversely applying calibrated hemostats to the nerve. For partial crush injuries, only half the diameter of the nerve was crushed. The entire diameter of the nerve was crushed for complete crush injuries. In the amplitude-based IONM, the healthy distribution for INS again closely recapitulates that of the baseline whereas ES healthy distribution broadens (Fig. 2a, b). This trend, however, is not as apparent for latency-based IONM in which INS exhibits a broader distribution (Fig. 2d, e). (Variance of baseline and healthy condition values is thoroughly examined in a subsequent section). Unlike with transection injuries, INS is more sensitive to partial and complete crush injuries. For both amplitude- and latency-based IONM, INS exhibits over a two-fold increase in sensitivity for the partial crush condition (Fig. 2c, f). In full crush experiments, INS successfully detected all damaged nerves (Fig. 2b, e) whereas ES failed to recognize these injuries in ~ 20% of the trials (Fig. 2a, d). Similar to the partial transections, INS fails to recognize partial crush injuries when upstream regions of undamaged axons were stimulated (Fig. 2b). This was again confirmed by translating the INS probe to fascicles that were damaged distally from the point of stimulation and observing a loss in amplitude and/or increase in latency (Fig. S1b). The distinct clustering seen in the patrial crush condition throughout Fig. 2 and subsequent figures results from separate experiments producing especially consistent amplitudes and latencies. This consistency is likely due to a combination of the positioning of the probe with respect to the injury and maintaining a stable degree of contact between the nerve and stimulation electrode for ES or maintaining a constant distance between the nerve and optical fiber for INS. Overall, crush injuries are more successfully recognized using amplitude rather than latency.

### Infrared neural stimulation surpasses electrical stimulation in stretch injury detection

**Figure 3:**
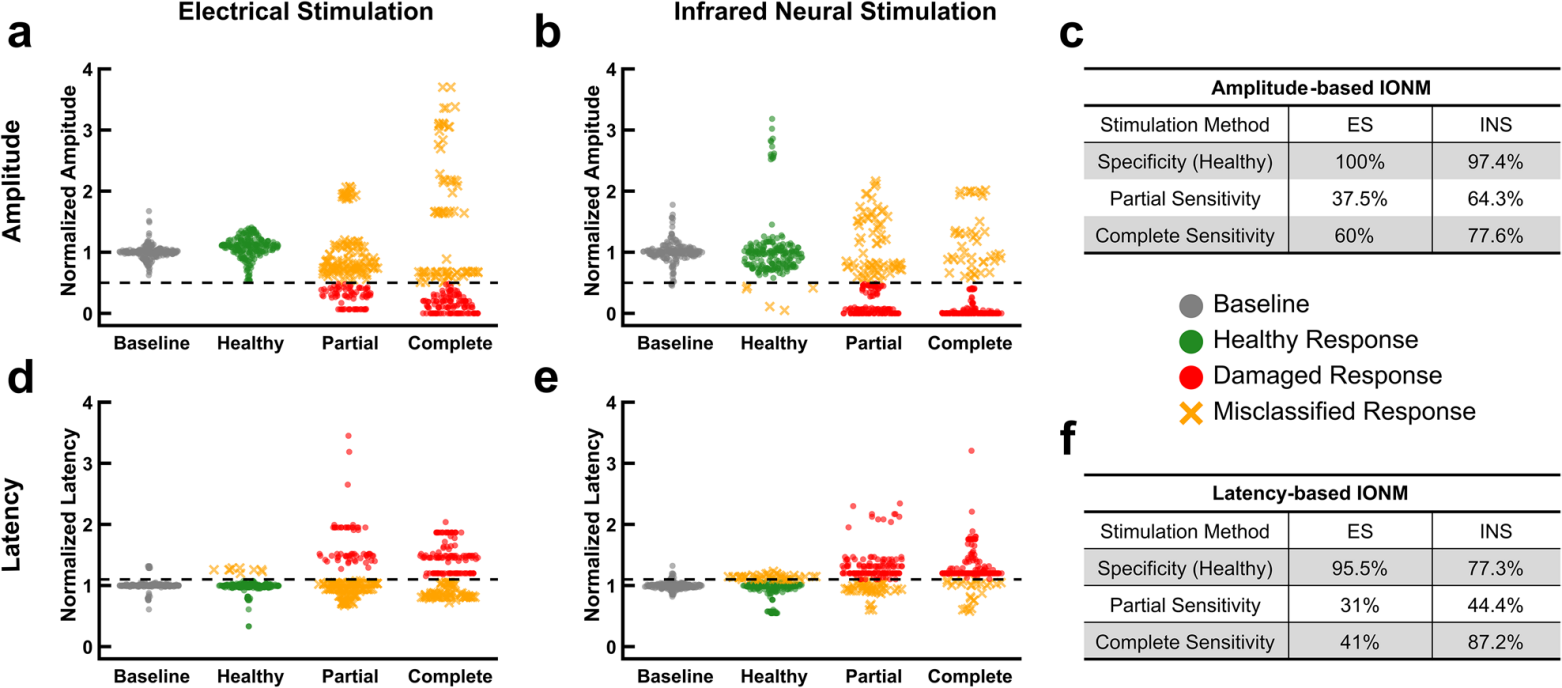
Stretch injuries are revealed with similar efficacy using both electrical and infrared neural stimulation. (**a**) Normalized CMAP amplitudes resulting from ES in baseline, healthy, partial stretch, and complete stretch conditions. Black dashed line represents the amplitude damage threshold (50% decrease). (**b**) Normalized CMAP amplitudes resulting from INS in baseline, healthy, partial stretch, and complete stretch conditions. (**c**) Sensitivity and specificity for the amplitude-based IONM approach to stretch injuries. (**d**) Normalized CMAP latencies resulting from ES in baseline, healthy, partial stretch, and complete stretch conditions. Black dashed line represents the latency damage threshold (10% increase). If no CMAP was evoked latency was set to 1.2 for ease of visual interpretation. (**e**) Normalized CMAP latencies resulting from INS in baseline, healthy, partial stretch, and complete stretch conditions. Note: Six normalized latency outliers in the partial stretch condition exceeded 4 (12–97) and were not plotted for better visualization. (**f**) Sensitivity and specificity for the latency-based IONM approach to stretch injuries. Specificity in all cases was calculated using the ‘Healthy’ category of responses. All data is normalized to the mean baseline values for each individual nerve. $n = 10$ nerves for all data sets (5 rats).

Prior to stretching the nerve, two marks ~ 2 mm apart were made on the nerve of interest using a surgical ink marker and the distance between the marks was measured using calipers. Stretch injuries were then induced using hook electrodes mounted to micro-manipulators. After putting the nerve in tension with the micro-manipulators, the distance between the marks was remeasured to calculate the strain. Partial and complete stretch injuries had an average strain of 8.6 and 13.4% respectively. For the partial stretch injury, strains of ~ 8% were chosen as multiple studies found strains between 5 and 10% to be the threshold for functional deficits resulting from stretch injuries^52–54^. Similarly, for the complete stretch condition, strains above 10% were selected since 10% strains had been previously shown to exceed the nerve’s mechanical tolerance^52^. INS yields a higher sensitivity to both partial and complete stretch than ES using both latency- and amplitude-based IONM (Fig. 3c, f). In particular, INS achieves a twofold increase in sensitivity in detecting complete stretch injuries using latency. The difference in sensitivities between the two modalities, however, is not as significant overall as compared with transection and crush injuries. Following stretch injuries, nerves often exhibited greater CMAP amplitudes than observed at baseline (Fig. 3a, b). The specificity of INS appears to suffer using latency-based IONM compared to ES (Fig. 3d, e). Observing the discrepancies in amplitude and latency values in undamaged nerves (i.e. between the baseline and healthy conditions) for both ES and INS, the baseline variance were examined.

### Infrared neural stimulation provides more consistent amplitude and latencies in undamaged nerves

**Figure 4:**
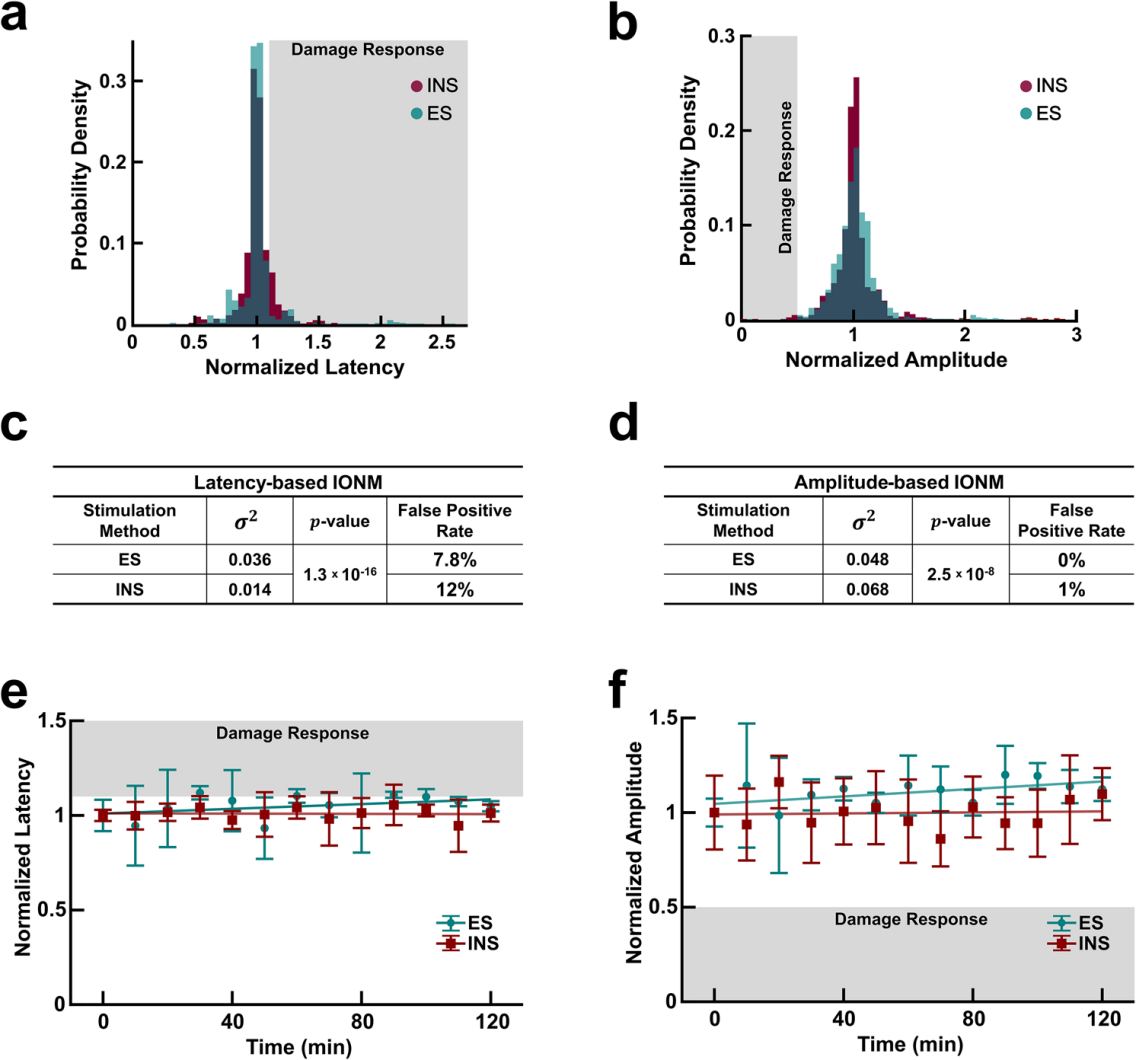
Electrical stimulation and infrared neural stimulation produce consistent latencies and amplitudes in undamaged nerves over extended periods of time. (**a)** Probability density function of normalized latencies produced in undamaged nerves resulting from ES (teal) and INS (red). Grayed region represents latency responses that exceed the damage threshold (10% increase in latency). (**b**) Probability density function of normalized amplitudes evoked in undamaged nerves resulting from ES and INS. Grayed region represents amplitude values that fall below the damage threshold (50% decrease in amplitude). (**c**) Statistical analysis of latency variance and false positive rate (**d**) Statistical analysis of amplitude variance and false positive rate. (**e**) Time course of normalized latency values over two hours produced by ES and INS. All values normalized to the mean at $t{ } = { }0\;{\text{min}}$. Linear fitting performed on all data for ES ($R^{2} = 0.16$) and INS ($R^{2} = 0.01$). (**f**) Time course of normalized amplitude values over two hours produced by ES and INS. All values normalized to the mean at $t{ } = { }0\;{\text{min}}$. Linear fitting performed on all data for ES ($R^{2} = 0.33$) and INS ($R^{2} = 0.004$). $n = 30$ nerves for all distribution graphs [**a**–**d**; 15 rats total]; $n = 3$ nerves and rats for time course plots (**e**–**f**).

Baseline and healthy data across all experiments were pooled to compare the variability in latency and amplitude of undamaged nerves (Fig. 4a, b). The probability density function of latency and amplitude in undamaged nerves for both ES and INS is not drawn from a normal distribution ($p = 0$ for all cases). Consequently, an Ansari-Bradley test was employed to test for equal variances (i.e. $\sigma^{2}$). In undamaged nerves, the test showed that ES and INS latencies possess unequal variances with INS exhibiting a smaller variance (Fig. 4c). ES produces a lower false positive rate (FPR) of 7.8% compared to 12% FPR of INS. Similarly for amplitude, ES and INS again have statistically distinct variances with ES having a significantly smaller standard deviation (Fig. 4d). Despite having unequal variances, both ES and INS share comparable FPR for amplitude. The FPRs for ES and INS were minimal at 0 and 1% respectively. In addition to examining baseline variability, the presence of any time dependent variance was also investigated.

Undamaged nerves were routinely stimulated using ES and INS for 2 hours while changes in latency and amplitude were monitored as seen in Fig. 4e and f. The 2 hour period was chosen to correspond not only with the maximum experiment duration but also exceed the average duration of a conventional thyroidectomy (~ 94 min)^55^. The magnitude of the latency values for both techniques remained largely constant over the course of the 2 hours (Fig. 4e). When all data points from the latency time course are examined, both INS and ES have nearly identical FPR of 0 and 0.5% respectively. In examining the time course of evoked amplitudes, ES and INS yielded the same FPR of 0.26%.

Given that INS offers comparable if not superior consistency to ES in addition to providing higher sensitivities, the performance of INS in conjunction with a clinical IONM system was then evaluated to ensure comparable efficacy and ease of integration.

### INS is readily incorporated into existing clinical IONM systems without loss of efficacy

**Figure 5:**
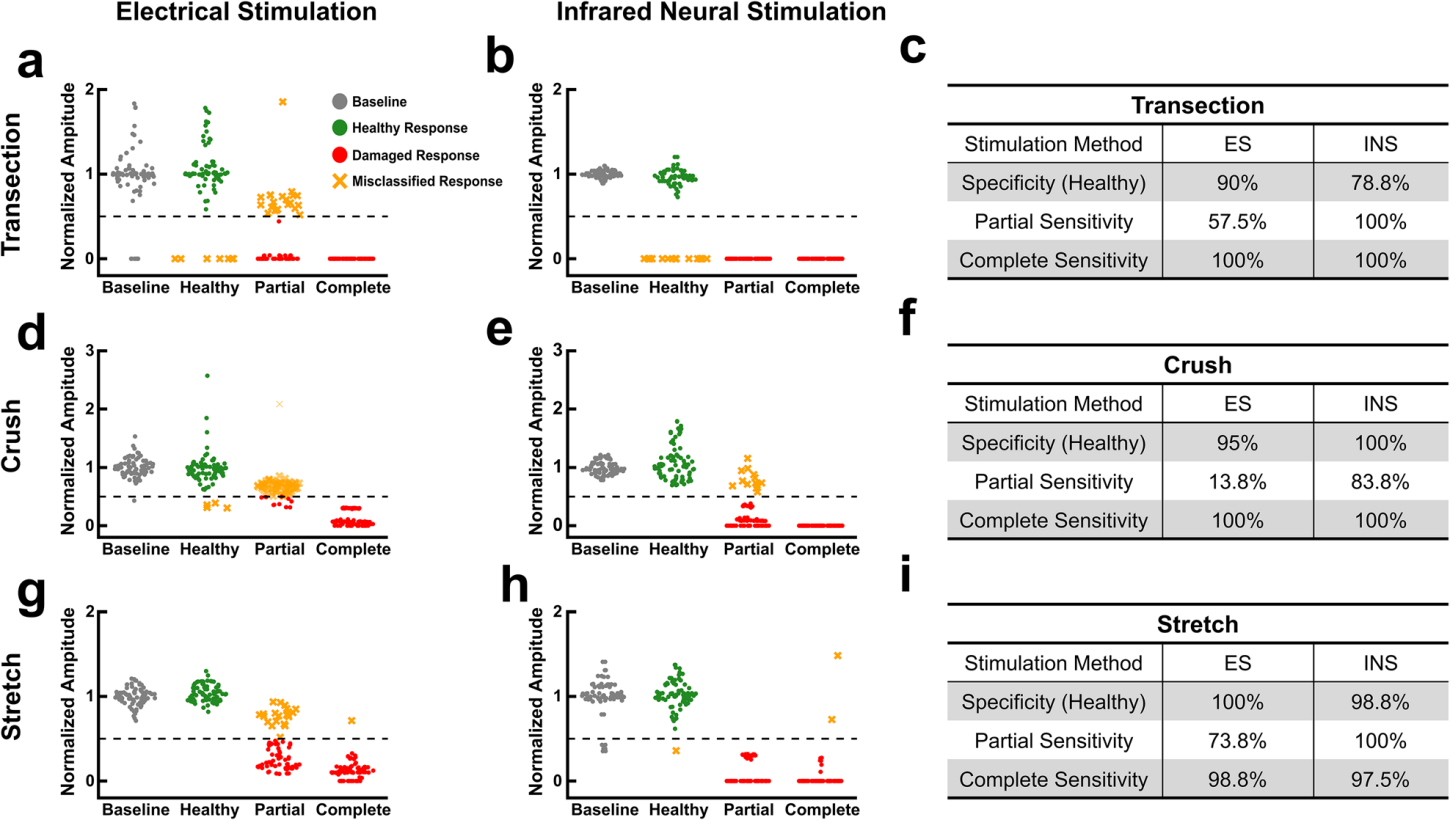
INS can be integrated in to existing clinical IONM systems while maintaining performance. (**a**) Normalized CMAP amplitudes resulting from ES in baseline, healthy, partial transection, and complete transection conditions. Black dashed line represents the amplitude damage threshold (50% decrease). (**b**) Normalized CMAP amplitudes resulting from INS in baseline, healthy, partial transection, and complete transection conditions. (**c**) Sensitivity and specificity for the amplitude-based IONM approach to transection injuries. (**d**) Normalized CMAP amplitudes resulting from ES in baseline, healthy, partial crush, and complete crush conditions. (e) Normalized CMAP amplitudes resulting from INS in baseline, healthy, partial crush, and complete crush conditions. (**f**) Sensitivity and specificity for the amplitude-based IONM approach to crush injuries. (**g**) Normalized CMAP amplitudes resulting from ES in baseline, healthy, partial stretch, and complete stretch conditions. (**h**) Normalized CMAP amplitudes resulting from INS in baseline, healthy, partial stretch, and complete stretch conditions. (**i**) Sensitivity and specificity for the amplitude-based IONM approach to stretch injuries. Specificity in all cases was calculated using the ‘Healthy’ category of responses. All data is normalized to the mean baseline values for each individual nerve. $n = 4{ }$ nerves for all data sets (2 rats).

**Figure 6:**
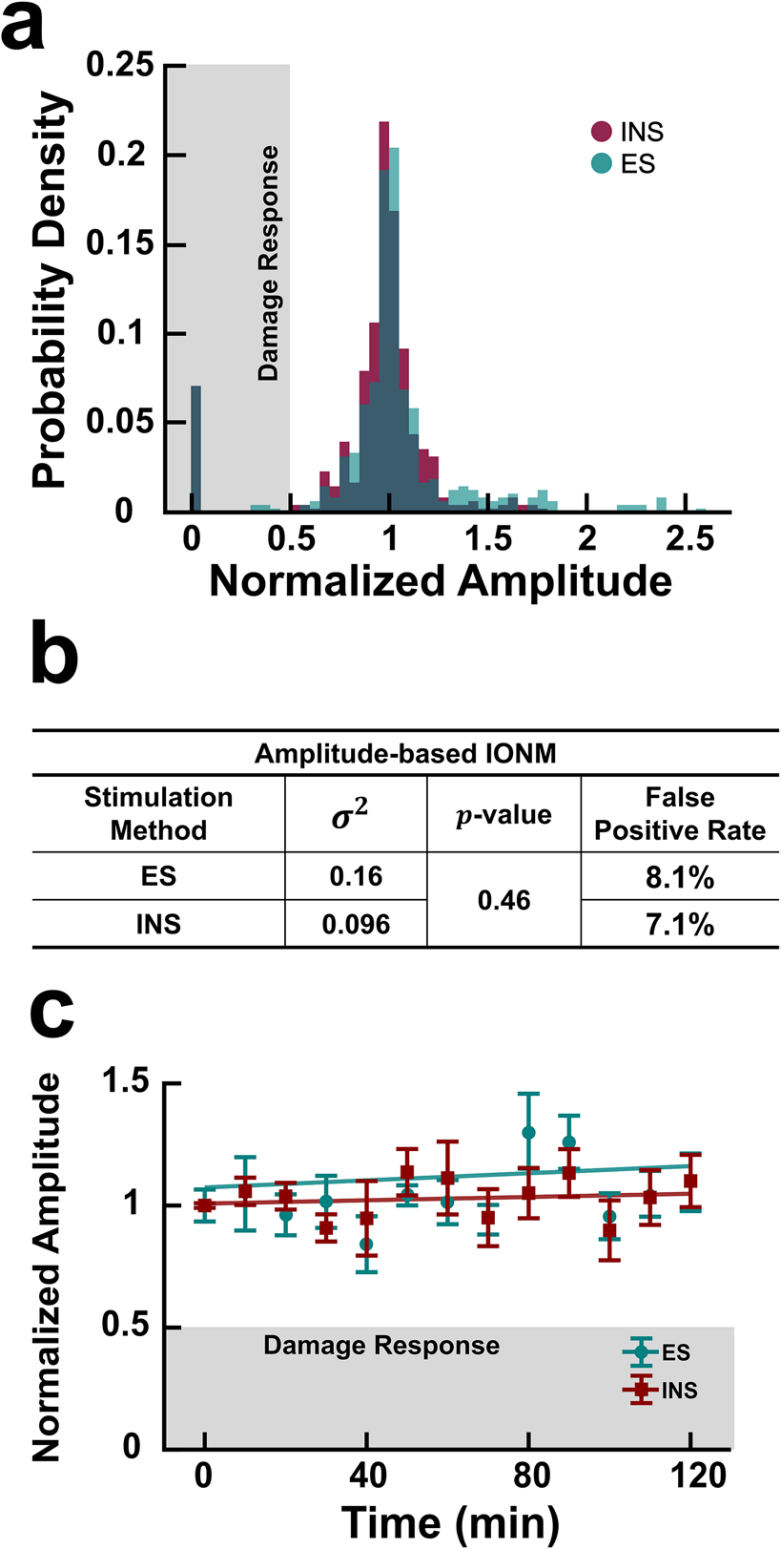
Electrical and infrared neural stimulation evoke consistent amplitudes over time as measured by clinical IONM system. (**a**) Probability distribution of normalized amplitudes evoked in undamaged nerves resulting from ES (teal) and INS (red) using a clinical IONM system. Grayed region represents amplitude values that fall below the damage threshold (50% decrease in amplitude). (**b**) Statistical analysis of amplitude variance and false positive rates. (**c**) Time course of normalized amplitude values over two hours produced by ES and INS. All values normalized to the mean at $t{ } = { }0\;{\text{min}}$. Linear fitting performed on all data for ES ($R^{2} = 0.131$) and INS ($R^{2} = 0.026$). $n = 12$ nerves for all distribution graphs [**a**–**b**; 6 rats total]; $n = 3$ nerves and rats for time course plot in (**c**).

Using a Medtronic NIM-Response 2.0, the same degrees and types of nerve injury were investigated to compare INS and clinical ES without any modifications to the system (Fig. 5). Since INS does not produce a stimulation artifact, latencies could not be accurately measured using the NIM 2.0. Hence, only amplitude-based IONM was examined using the clinical system. Similar to the benchtop system, INS detects all partial transections almost doubling the sensitivity of ES (Fig. 5a–c) similar to what was observed using the benchtop system. As expected, both techniques accurately identified all complete transections. The specificity of ES, however, was substantially higher than that of INS in transection experiments which is more thoroughly explored in Fig. 6. When examining crush injuries, INS vastly outperformed ES in identifying the onset of partial crush with a sensitivity of 83.8 and 13.8% respectively while both correctly classified all full crush CMAPs as damaged (Fig. 5d–f). Trials in which INS failed to detect partial crush injuries were again due to its spatial selectivity (see Fig. S1b). For crush injuries, both INS and ES shared nearly equivalent specificities. For stretch injuries, INS surpasses or matches the sensitivity of ES as seen with the benchtop system. In addition to comparable specificities, INS and ES also produced practically equal sensitivities to complete stretch injuries (Fig. 5g–i). The sensitivity of ES to partial stretch injury did suffer while INS maintained a consistent level of sensitivity for both the partial and full conditions as was the case using the benchtop system.

Baseline and healthy amplitudes across each trial using the clinical IONM system were combined to analyze the variance of both techniques in undamaged nerves. The probability density function of these amplitudes is depicted in Fig. 6a. The amplitudes in undamaged nerves for both ES and INS were not normally distributed ($p = 0$ for all cases). Subsequent testing for variance equivalence revealed the two distributions do not have significantly different variances (Fig. 6b). Though the two techniques derive from similar distributions, INS still produced a smaller variance than ES. In addition to having statistically equivalent variances, the FPRs for INS and ES were 7.1 and 8.1% respectively. Changes in variance were also examined over time.

Undamaged nerves were routinely stimulated using ES and INS for 2 hours while changes in amplitude were monitored using a clinical IONM system (Fig. 6c). Overall, amplitude values remained relatively consistent over the course of the 2hour period. Both techniques had an equivalent FPR of 2% across that time frame.

## Discussion

Iatrogenic nerve injury (INI) is a dreaded complication amongst surgeons across disciplines that detrimentally affects patient, provider, and hospital. In procedures where INIs are readily possible or result in severe complications, IONM has become standard practice. During IONM, physiological signals from the nerve’s effector are checked for signs of damage after being electrically stimulated. Multiple studies have shown that IONM has reduced the incidence of INIs^15,20–23^. Since current IONM relies on ES to generate the necessary evoked potentials, IONM is limited by the inherent limitations of ES namely: current spread, the presence of ES artifacts, and tissue contact which can lead to erroneous results. Due to its high spatial specificity as well as artifact and contact free nature, INS overcomes many of these obstacles and has been touted as both a promising alternative to ES and means to improve IONM. Most studies to date, however, have shown that INS can elicit relevant signals safely, stopping short of providing evidence of INS’ clinical value. In this study, INS was directly compared to ES using both benchtop and clinical IONM systems before and after different types of nerve injury in vivo in an animal model. Using the clinical thresholds for nerve damage detection and examining the three most reported types of INI, our results show that INS surpasses ES in partial injury detection while maintaining similar efficacy and consistency for complete injuries. In the case of partial injuries, INS vastly outperforms ES.

For partial transections, INS exhibited sensitivities over two times higher than ES using both amplitude- and latency-based IONM. This improvement also held when using the clinical IONM system. Though the sensitivity of ES did improve to 58% when using the clinical system, the sensitivity of INS, however, was almost twice as high at 100%. Hence, INS offers tremendous improvement over ES which produced sensitivities as low as 20%. With sensitivities 40–70% higher than ES, this trend was also apparent for partial crush injuries using both the benchtop and clinical systems. The poor performance of ES in detecting partial crush and transections is likely due to current spread during which unconfined electrical stimulus activates surrounding intact axons still capable of generating adequate CMAPs. Due to its innate spatial selectivity, INS is more sensitive to partial transection and crush injuries. In recruiting a smaller population of axons, damage to fewer axons will result in a more discernible change in the evoked response. This spatial selectivity, however, can also lead to false negatives as seen in Fig. 1b (and again in Figs. Fig. 2b and d) when nondamaged portions of the nerve are stimulated (also see Fig. S1). Hence, looking towards clinical translation, the ability to target individual fascicles or specific portions of nerves will be essential for optimal efficacy and can be easily achieved using multifiber arrays or additional optics. In contexts where the spatial specificity of INS is not desired or larger targets are of interest, the spatial precision of INS can be modified. By adjusting the wavelength and spot size used for stimulation, the stimulated volume can be tailored to specific applications. For partial stretch injuries, the difference in sensitivities between INS and ES was not as stark as with transection and crush.

Using amplitude, there was only 26% difference in sensitivities of INS and ES for partial stretch injuries in both the benchtop and clinical system. INS did attain a sensitivity of 100% using the clinical system, however. Using latency, the difference was only 13% with INS achieving the higher sensitivity of 44%. In general, both techniques poorly detected the presence of partial stretch injuries. This may be attributable to the fact that the strains applied for partial stretch injuries were in most cases recoverable and possibly insufficient to drastically affect amplitude and latency of the evoked CMAPs which has been observed in previous studies^52,56^. The smaller difference in sensitivity between INS and ES may also be a consequence of the entire nerve being stretched rather than a fraction of its diameter. Hence, the spatial precision of INS does not contribute to its diagnostic accuracy. Both modalities are probing stretched axons which appears to be a difficult type of damage to classify using latency and/or amplitude. Moreover, with stretch injuries, some amplitudes evoked after stretching were substantially higher than those at baseline (Fig. 3a, b). Other studies have reported similar findings and provide evidence that stretch injuries increase excitability as well as generate greater amplitude CMAPs especially during recovery periods as short as 3 minutes^57^. Thus, it is possible some nerves had sufficient time to recover between stretching and stimulation. It should also be noted that the stretch injuries inflicted here cannot be completely decoupled from the trauma caused by the hooks used to stretch the nerve. The IONM results for both stimulation techniques after stretch, however, are quite distinct from both transection and crush injuries as stated previously. Since the hooks would likely cause a compression or crush injury, this suggests that a different type of damage (i.e. stretch) is occurring. Despite both techniques leaving room for improvement in the detection of partial stretch injuries, INS dependably identified more injuries than ES using both nerve monitoring systems and metrics (i.e. amplitude and latency). In moving from partial to complete forms of damage, however, the efficacy of INS and ES were largely on par.

As expected, ES and INS correctly identified all complete transections using both the benchtop and clinical systems. For complete crush injuries, the performance of the two techniques was also comparable. With the benchtop system, however, INS correctly classified every evoked response as damaged while ES reached a sensitivity of ~ 80% using amplitude and latency-based IONM. Both techniques attained sensitivities of 100% using the clinical IONM system. Compared to the partial condition, the sensitivity for both ES and INS increase slightly for complete stretch injuries using the benchtop system. Nonetheless, INS again produced higher sensitivities than ES except when using the clinical system which yielded nearly equivalent sensitivities. For each complete injury type, INS often exceeded or at the very least matched the efficacy of ES using both IONM systems and approaches. Additionally, INS consistently achieved a higher sensitivity than ES for both degrees of severity, IONM systems, and approaches. While the efficacy of INS surpassed that of ES, amplitude-based IONM also generally provided more accurate classification than latency-based IONM.

Taken as a whole, latency-based IONM regularly underperformed compared to the amplitude-based approach. Latency-based IONM only bettered amplitude-based once in recognizing complete stretch injuries using INS. Moreover, for complete transections and crush injuries, latency-based sensitivities matched that of amplitude-based exclusively when no CMAPs were evoked. Although latency was only measured with the benchtop system, these results seem to suggest that amplitude-based IONM offers a more robust and accurate indication of nerve health and functionality. This may also account for reason many surgeons only utilize amplitude-based IONM rather than latency alone or a combination of the two. In addition to investigating the sensitivity of INS during IONM, the specificity and consistency of INS-induced amplitudes and latencies was also analyzed over time and across systems.

The statistical analysis of ES- and INS-induced latency distributions in undamaged nerves revealed that both have unequal variances (Fig. 4c). Of the two, INS had the smaller variance and consequently a standard deviation 7% lower than that of ES. Since the latency damage threshold is defined as a 10% increase from baseline, this suggests that having a smaller standard deviation even by 7% could improve nerve damage detection and reduce the risk of false positives. Extended monitoring of undamaged nerves over a period of two hours showed that INS and ES induced latencies had equal FPR over the duration (Fig. 4c). INS did, however, exhibit a higher FPR than ES in the short term (< 10 min after baseline acquisition) based on the data from nerve injury trials. Overall, this suggests that INS produces comparable if not more consistent latencies over time than ES. This trend was also observed in INS- and ES-induced amplitudes.

Both ES and INS amplitude distributions also have statistically different variances for the bench top system with ES has having a smaller overall standard deviation by 4% (Fig. 4d). In relation to the 50% decrease in amplitude damage threshold, this difference is variance is insignificant. Accordingly, both techniques have statistically equivalent variances when using the clinical IONM system. Using both systems, each technique had FPRs differing less than a percent across all considered time frames (Figs. 4d, 6b). Variability in ES amplitude data is likely due to slight variations in electrode’s contact with the nerve (Fig. 1a) while the lower variability of INS mediated CMAPs is possibly due to the fact INS is non-contact. Hence, the efficacy of INS may be less susceptible to probe placement and manipulations of the surgical field as long as its spatial selectivity is well managed. Since water absorption of infrared light is the driving mechanism underlying INS, the primary source of variability in INS data is likely due to changes in tissue hydration. Taken together, INS provides more consistent amplitudes and latencies in undamaged nerves and provides comparable if not less variability than ES.

By integrating INS into a clinical IONM system, we also took the first step to show that the benefits INS offers are readily translatable to existing IONM systems. The results confirm that INS is easily incorporated into clinical IONM systems already in use during surgery without a loss in efficacy. This provides a clear path for INS into the operating room with minimal disruption to current surgical workflows. If quantification of latency is desired, additional modifications to current clinical IONM would need to be made to allow for accurate recordings of both the optical stimulus and evoked signal. Given that latency-based IONM seems to frequently provide erroneous classifications and some surgeons chose to rely solely on amplitude, these modifications may not be in high demand.

Using the clinical thresholds for nerve damage detection, we have demonstrated that INS, a safe and proven neurostimulation method, is more sensitive to partial forms of damage than clinical ES and exhibits equal if not superior sensitivity to more severe injuries. The enhanced sensitivity of INS is largely due to its high degree of inherent spatial selectivity. With improved sensitivity to nerve injury, surgeons could be alerted to the onset of damage earlier preventing further trauma and enabling timely interventions. Moreover, INS largely yields more consistent and reliable latencies and amplitudes in undamaged nerves. Hence, in surgery, INS has the potential to provide more consistent, reliable values and clearer, more accurate indications of nerve damage. Able to be readily integrated into current clinical IONM systems, the findings of this study substantiate the clinical value of INS for IONM and propose a simple means to improve surgical outcomes by sparing both patients and surgeons from the adverse effects of INIs.

## Methods

### Animal preparation

All experiments were conducted at the Vanderbilt Biophotonics Center in adherence to protocols approved by the Vanderbilt Institution of Animal Care and Use Committee (IACUC) and are reported in accordance with ARRIVE guidelines where applicable. All methods were performed according to the relevant ethical guidelines and regulations as approved by the Vanderbilt IACUC. The surgical preparations used here have been described elsewhere in detail^37^. Briefly, adult male and female Sprague–Dawley rats (250–300 g) were anesthetized and maintained under sedation using isoflurane ($n=30$ total). To expose the sciatic nerve and its trifurcations, a ~ 3 cm incision was made on the lateral side of the leg extending from the gluteus to popliteal region using a split-muscle technique. Room temperature sterile saline was routinely applied to the nerve throughout experiments to maintain tissue hydration and prevent desiccation.

### Benchtop electrophysiology

Experiments utilizing research grade equipment were performed with a modular data acquisition system (MP100, Biopac Systems Inc., Santa Barbara, California) to simultaneously record evoked CMAPs and INS or ES triggering. This enabled accurate calculation of latencies values resulting from both INS and ES. For experiments on the common peroneal and tibial nerve, paired, bipolar subdermal needle electrodes (Medtronic Xomed, Jacksonville, Florida) were placed in either the tibialis anterior or soleus muscle respectively to record evoked activity. Both a subdermal grounding electrode and stimulus return were also inserted into the foot on the ipsilateral leg with the ground more proximal to the location of the applied stimulus. Evoked signals were sampled at a rate of 6500 Hz, amplified 1000x, and bandpass filtered from 0.05 to 5000 Hz with a differential amplifier (DA 100C, Biopac Systems Inc., Santa Barbara, California).

### Clinical electrophysiology

A NIM-Response 2.0 (Medtronic, Minneapolis, Minnesota) was used in experiments demonstrating INS compatibility with existing clinical IONM systems. Just as with the benchtop system, bipolar subdermal needle electrodes were placed in either the tibialis anterior or soleus muscle. Additional subdermal electrodes were used for grounding and stimulus return and placed in the ipsilateral foot. NIM-Response 2.0 bandpass filtering was internally fixed by the manufacturer at 100–2000 Hz. The electrical stimulation artifact delay on the NIM-Response 2.0, used as means to work around the stimulation artifact, was set to 3.1 ms.

### Infrared neural stimulation

A 1450 nm diode laser (Capella, Lockheed Martin-Aculight, Bothell, Washington) coupled to a 400 µm core bare fiber (NA = 0.22; Ocean Optics, Dunedin, Florida) was used for all INS experiments. The optical fiber was positioned orthogonal to the nerve surface using a micromanipulator (World Precision Instruments, Sarasota, Florida). In accordance with previous optimization studies, diode current was adjusted to deliver radiant exposure between 1.4 and 1.6 J/cm^2^ at a pulse width of 500 μs^37^. The stimulation radiant exposure was determined by incrementally increasing the diode current until a muscle twitch was achieved for every delivered pulse. Pulse trains lasting 10 s at a repetition rate of 2 Hz were employed for every nerve monitoring trial to minimize thermal superposition^58^. The optical fiber was positioned to not be in contact with the tissue at distance of ~ 120 μm such that the average spot size at the tissue was 503.6 ± 16 μm (1/e^2^ diameter) as measured by an infrared beam profiler (BP209-IR2, Thorlabs, Newton, New Jersey) and validated using the knife-edge technique^59^.

### Electrical stimulation

A standard Prass monopolar stimulator probe (Medtronic Xomed, Jacksonville, Florida) was used for all experiments. When using the benchtop system, monophasic, square pulses with currents < 1 mA were used to evoke CMAPs. To match INS stimulation parameters, ES nerve monitoring trials with the benchtop system were performed with a pulse width of 500 $\mu s$ at frequency of 2 Hz. Trials using the NIM-Response 2.0 system the pulse width was set to 100 µs and the frequency to 4 Hz while maintain a stimulus < 1 mA. The ES threshold was determined by incrementally increasing the applied current until a muscle twitch was induced for each delivered stimulus.

### Nerve injury

Three types of INI were investigated in this study: transection, crush, and stretch. Each was examined at two degrees of severity: a partial and complete form. Transection injuries were inflicted by cutting through approximately half of and the entirety of the nerve’s diameter for the partial and complete forms of damage respectively. Partial transection injuries were inflicted using a 3D printed nerve cutting guide fitted with a semicircular nerve notch to hold the nerve in place. One guide had nerve notch with a diameter equal to the average diameter of the rat common peroneal nerve (0.4 mm) and other the average diameter of the tibial nerve (0.63 mm). Both guides were fitted with a blade tract that directed the razor blade to make a transverse cut through half the diameter of the respective nerve.

Crush injuries were made perpendicular to the axis of the nerve using Kelly hemostats with a closing force of 1.12 N. Complete crush injuries were made by crushing the entire diameter of the nerve. For partial crush injuries, only half of the diameter of the nerve was crushed. For both partial and complete crush injuries, the hemostats were left clamped to the nerve for 10 s duration to allow stable compression.

For stretch injuries, the nerve was put into tension using hook electrodes mounted to a micromanipulator. To measure strain, two dye markers were placed on the surface of the nerve before insult, and the distance between the two was measured with calipers (0.1–1 cm). After the nerve was stretched using the micromanipulator, the distance between the dye markers was measured again and used to calculate the induced strain. Partial stretch injuries resulted in an average induced strain of $8.62 \pm 1.6$ and $13.4 \pm 3.6\%$ for complete stretch injuries. There was no difference in the pulling strength between partial and complete conditions. The induced strains were chosen based on the work of Rickett et al.^52^ With respect to partial stretch injuries, Rickett et al. observed the minimum threshold for functional deficit after a nerve was stretched between 5 and 10%. This study was also supported by Driscoll et al. and Li and Shi who also reported functional deficits at 8.8 and 8.3% respectively^53,54^. Similarly, for the complete stretch condition strains greater than 10% were chosen as Rickett et al. observed that the mechanical tolerance of the nerve was exceeded above 10%.

### Data analysis

As the current clinical standard, a ≥ 50% loss in baseline amplitude and a ≥ 10% increase in the baseline latency served as the thresholds for neural damage detection^50,51^. Amplitude was defined as the difference between the maximum and minimum of the evoked response to eliminate the need for any baseline corrections in the recordings. Latency was defined as the duration from the peak of the stimulus to the peak of the evoked response. For all trials, “Healthy”, “Partial”, and “Complete” amplitudes and latencies were normalized to the mean of the corresponding baseline values from individual experiments. In benchtop experiments, only trials that resulted in a clear separation of the ES artifact and evoked CMAP were considered in data analysis in order to accurately quantify amplitude and latencies.

### Statistical analysis

All datasets were tested for normality using a Kolmogrov-Smirnov test. As most distributions were not normal, equivalence of variance was evaluated using an Ansari-Bradley test.

All sensitivity calculations were made utilizing CMAP amplitudes and latencies obtained after nerve injury using the standard formula:

$$Sensitivity = \frac{True\;Positives}{{True\;Positives + False\;Negatives}}$$

where true positives correspond to values correctly classified as damaged responses (i.e. an amplitude < 50% or a latency > 110% of their respective baseline values) and false negatives correspond to values incorrectly classified as healthy responses (i.e. an amplitude > 50% or a latency < 110% of their respective baseline values). Since these values were obtained after the nerve was injured, the true positives represent the accurate classification of the nerve as damaged while the false negatives inaccurately indicate that the nerve is undamaged/healthy. Similarly, specificity was calculated using CMAP amplitude and latencies elicited after baseline acquisition and prior to nerve injury (i.e. while the nerve remained healthy and undamaged). Specificity was calculated using the standard formula:

$$Specificity = \frac{True\;Negatives}{{True\;Negatives + False\;Positives}}$$

where true negatives correspond to values correctly classified as healthy responses (i.e. an amplitude > 50% or a latency < 110% of their respective baseline values) and false positives correspond to values incorrectly classified as damaged reponses (i.e. an amplitude < 50% or a latency > 110% of their respective baseline values). Since these values were obtained before the nerve was injured, the true negatives represent the accurate classification of the nerve as undamaged and healthy while the false positives inaccurately indicate that the nerve is damaged. This calculation was identical for both partial and complete forms of injury, amplitude and latency metrics, benchtop and clinical IONM systems, and across all three injury types.

All false positive rates were calculated using the amplitude and latencies obtained from undamaged nerves and the standard formula:

$$False\;Positive\;Rate = \frac{False\;Positives}{{False\;Positives + True\;Negatives}}$$

Since the false positive rate is only calculated in healthy, undamaged nerves, false positives correspond to amplitudes and latencies that incorrectly indicated the nerve was damaged (i.e. amplitudes < 50% or latencies > 110% their respective baseline values). Accordingly, true negatives correspond to amplitudes and latencies that correctly indicated that the nerve was undamaged (i.e. amplitudes > 50% or latencies < 110% their respective baseline values).

## Supplementary Information

Supplementary Information.

## Data Availability

The datasets generated and analyzed during the current study are available from the corresponding author on reasonable request.
