# Supplementary material for: Infrared neural stimulation markedly enhances nerve functionality assessment during nerve monitoring
Source: Sci Rep. 2023 Mar 16;13:4362. doi: 10.1038/s41598-023-31384-3 (PMC10020565; doi:10.1038/s41598-023-31384-3)
Supplement: Supplementary file 1 — Supplementary Information. [file 41598_2023_31384_MOESM1_ESM.pdf]

Supplemental Figures:

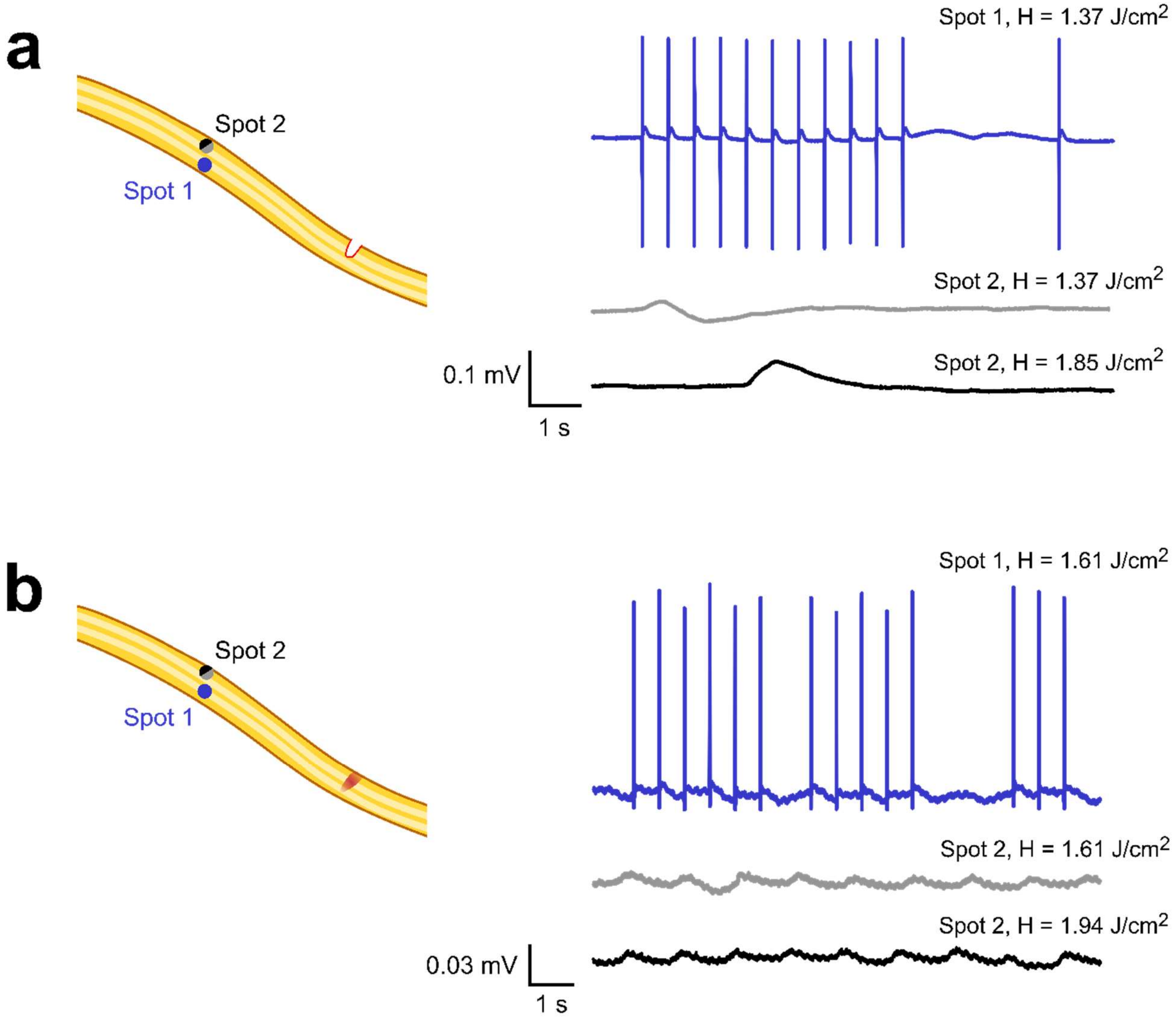

Figure S1. Nerve monitoring efficacy of infrared neural stimulation is dependent on its spatial selectivity. a) Illustration and representative CMAP traces from partially transected nerve. The blue trace and Spot 1 correspond to the stimulation of intact nerve fascicles resulting in a false negative (undamaged nerve). Black and gray traces correspond to upstream stimulation of damaged fascicles at the same radiant exposure as Spot 1 (gray) and at a higher radiant exposure (black). **b)** Illustration and representative CMAP traces from a partially crushed nerve. The blue trace and Spot 1 correspond to the stimulation of intact nerve fascicles resulting in a false negative. Black and gray traces correspond to upstream stimulation of damaged fascicles at the same radiant exposure as Spot 1 (gray) and a higher radiant exposure (black).
